# P-1307. AI-Optimized Assessment of Vaccination Strategies in Smallpox Reemergence: an LA Case Study

**DOI:** 10.1093/ofid/ofae631.1488

**Published:** 2025-01-29

**Authors:** Tristan Learoyd, John E Pittman

**Affiliations:** Emergent BioSolutions, Redcar, England, United Kingdom; Emergent BioSolutions, Redcar, England, United Kingdom

## Abstract

**Background:**

The resilience of Los Angeles (LA) to smallpox was evaluated using a modified Susceptible, Exposed, Infectious, Recovered, Hospitalized, Deceased (SEIRHD) model, which simulated its spread via an artificial intelligence (AI) mobility matrix and assessed different vaccination regimens (one dose primary series vs a two dose primary series) for outbreak control.

**Methods:**

The matrix combined data from travel surveys, transportation authorities, GPS caches, and population density estimates from LA County. Fifteen LA government districts plus a 30-mile radius city limit were considered for differential force of infection and mobility matrix variables. Transmission parameters, derived from a review of 20th-century epidemics, assigned 90% transmission to households and 80% to workplaces. Residual immunity against smallpox was set at 8.8%. Intensive care unit (ICU) capacity within 30 miles of LA city limits was set as 2,500 beds per the California Health and Human Services Open Data Portal. One and two dose ring vaccine regimens initiated at day 7 were assessed in 16 districts and as a dispersed city model to understand zonal resilience in eradication and if contagion exceeded ICU capacity limits via an R-programed scenario.

**Results:**

The naïve general scenario had an infection peak at day 73, with a hospitalization peak of 5,600,227 (day 80) of a susceptible population of 18,833,640. With a 50% effective one dose vaccine, contagion remained below the ICU threshold in the basic 30-mile radius model. However, in specific districts (1, 6-9, and 13-15), even a 95% effective one dose vaccine couldn’t maintain contagion below the ICU threshold according to the AI-assisted zonal model. Hypothetical one dose primary series vaccination regimens outperformed two dose regimens in both city-wide and zonal scenarios (P< 0.05). Zonal analysis based on housing conditions and mobility revealed that even with the most effective hypothetical one dose vaccine (95% efficacy after 7 days), 7 out of 15 LA district ICU thresholds were exceeded, indicating the importance of geospatial modelling.

**Conclusion:**

The model highlights the importance of zone-based evaluations in preparedness and the need for high-efficacy single-dose vaccinations in ring vaccination.

**Disclosures:**

**Tristan Learoyd, MPharm MA MSc MBA PhD MRPharmS**, Emergent: Employee **John E. Pittman, PharmD**, Emergent: Employee

